# Less and less–influence of volume on hand coverage and bactericidal efficacy in hand disinfection

**DOI:** 10.1186/1471-2334-13-472

**Published:** 2013-10-10

**Authors:** Günter Kampf, Sigunde Ruselack, Sven Eggerstedt, Nicolas Nowak, Muhammad Bashir

**Affiliations:** 1 Bode Science Center, Bode Chemie GmbH, Melanchthonstr. 27, 22525 Hamburg, Germany; 2 Institut für Hygiene und Umweltmedizin, Ernst-Moritz-Arndt Universität Greifswald, Walther-Rathenau-Str. 49a, 17475 Greifswald, Germany; 3 Development, Bode Chemie GmbH, Melanchthonstr. 27, 22525 Hamburg, Germany; 4 Scientific Affairs, Bode Chemie GmbH, Melanchthonstr. 27, 22525 Hamburg, Germany; 5 MicroBioTest Division of Microbac Laboratories, Inc, 105 Carpenter Drive, Sterling, VA, USA

**Keywords:** Alcohol-based hand rub, Hand coverage, Volume, Efficacy

## Abstract

**Background:**

Some manufacturers recommend using 1.1 mL per application of alcohol-based handrubs for effective hand disinfection. However, whether this volume is sufficient to cover both hands, as recommended by the World Health Organization, and fulfills current efficacy standards is unknown. This study aimed to determine hand coverage for three handrubs (two gels based on 70% v/v and 85% w/w ethanol and a foam based on 70% v/v ethanol) applied at various volumes.

**Methods:**

Products were tested at product volumes of 1.1 mL, 2 mL, 2.4 mL as well as 1 and 2 pump dispenser pushes; the foam product was tested in addition at foam volumes of 1.1 mL, 2 mL, and 2.4 mL. Products were supplemented with a fluorescent dye and 15 participants applied products using responsible application techniques without any specific steps but the aim of completely covering both hands. Coverage quality was determined under ultraviolet light by two blinded investigators. Efficacy of the three handrubs was determined according to ASTM E 1174-06 and ASTM E 2755-10. For each experiment, the hands of 12 participants were contaminated with *Serratia marcescens* and the products applied as recommended (1.1 mL for 70% v/v ethanol products; 2 mL for the 85% w/w ethanol product). Log_10_-reduction was calculated.

**Results:**

Volumes < 2 mL yielded high rates of incomplete coverage (67%–87%) whereas volumes ≥ 2 mL gave lower rates (13%–53%). Differences in coverage were significant between the five volumes tested for all handrubs (p < 0.001; two-way ANOVA) but not between the three handrubs themselves (p = 0.796). Application of 1.1 mL of 70% v/v ethanol rubs reduced contamination by 1.85 log_10_ or 1.60 log_10_ (ASTM E 1174-06); this failed the US FDA efficacy requirement of at least 2 log_10_. Application of 2 mL of the 85% w/w ethanol rub reduced contamination by 2.06 log_10_ (ASTM E 1174-06), fulfilling the US FDA efficacy requirement. Similar results were obtained according to ASTM E 2755-10.

**Conclusions:**

Our data indicated that handrubs based on 70% ethanol (v/v) with a recommended volume of 1.1 mL per application do not ensure complete coverage of both hands and do not achieve current ASTM efficacy standards.

## Background

Use of an alcohol-based handrub at specific times during patient care is strongly recommended by the World Health Organization (WHO) to ensure patient safety [1]. The consensus recommendation says, “Apply a palmful of alcohol-based handrub and cover all surfaces of the hands. Rub hands until dry.” The volume of handrub is not specified. User hand sizes vary, so proposing a specific volume might be difficult. Nevertheless, the volume must be large enough to “cover all surfaces of the hands”. In Europe, the proposed volume for hygienic hand disinfection was 3 mL for many years [2]. This volume was based on efficacy data from European Norm (EN) 1500 and on clinical practice experience that found that 3 mL of handrub resulted in hands that remained wet for approximately 30 s. Although data from EN 1500 on some products now shows that the same efficacy might be achieved in only 15 s [3], the European recommendation to keep hands wet for 30 s has not changed [4]. The main reason is that hand surfaces are poorly covered by shorter application times (e.g., 15 s) with almost all hands showing coverage gaps [5]. For more than 10 years, many countries have been using handrubs with a low alcohol concentration (i.e., 60%–70% ethanol) despite evidence that antimicrobial efficacy of this alcohol concentration is limited, even for applications of 3 mL for 30 s [6]. Smaller volumes are even less effective [7]. In addition, we see a trend from some manufacturers to recommend use of smaller volumes based on efficacy data generated mainly by ASTM E 1174 or ASTM E 2755 as well as EN 1500 [8]. These methods from the American Society for Testing and Materials (ASTM) and the European norms (EN) allow measuring the antimicrobial efficacy on artificially contaminated hands simulating practical conditions. This trend to smaller volumes is attractive for manufacturers to win tenders but the impact on patient safety should also be addressed carefully. In 2008, a volume of 2.4 mL was described as sufficiently effective by the FDA’s Tentative Final Monograph for Healthcare Antiseptic Products [9] for formulations based on 85% ethanol [10]. In 2012, a volume of 2 mL was described as sufficient for efficacy according to ASTM E 1174-94 for formulations containing 62% ethanol [8]; also in 2012, efficacy data for a single formulation based on 70% ethanol were presented for volumes as low as 1.1 mL [11]. The use of handrubs with low alcohol concentration in combination with small application volumes raises the concern that this combination might not meet the WHO recommendation “to cover all surfaces of the hands.” In addition, small volumes might not be sufficiently effective. For this reason, we studied the quality of hand coverage and efficacy of various application volumes of three commonly used handrubs.

## Methods

### Handrub preparations

Three commercially available handrubs were used: one was a gel based on 85% (w/w) ethanol (Sterillium Comfort Gel, Bode Chemie GmbH, Hamburg, Germany), one was a gel based on 70% (v/v) ethanol (Purell Advanced Instant Hand Sanitizer; Gojo Industries, Akron, OH, USA), and one was a foam based on 70% (v/v) ethanol (Purell Advanced Instant Hand Sanitizer Foam; Gojo Industries). For hand coverage experiments, each product was supplemented with 1.96% fluorescent dye (Visirub, Bode Chemie GmbH, Hamburg, Germany).

### Evaluation of hand coverage

#### Test subjects

Each application variation was performed by 15 test participants (office workers, laboratory technicians, chemists and purchasers). None were healthcare professionals. Non-healthcare workers were chosen so that the participants would not routinely apply alcohol-based handrubs, for better assessment of the practicality of the application procedure. As soon as hands felt dry, application was stopped and the time recorded. From all participants, gender, age and dominant hand were obtained. Ethical approval was not considered necessary for this part of study.

#### Product application

All three formulations were applied at volumes of 1.1 mL, 2 mL, and 2.4 mL; and 1 or 2 dispenser pushes. The manufacturers offer for each product an automatic dispenser which allows an accurate volume to be dispensed but also a manual pump dispenser which was used for each product in our study. The applied volume of the foam product was based on liquid volume, but conversion to foam resulted in larger cubic volumes. Foam was also applied as 1.1 mL, 2 mL and 2.4 mL, measured as foam volume, resulting in a total of 18 variations. All variations were blinded with a number. The 18 procedures were performed in a random sequence. “Responsible application” was recommended for each variation which allows the participant to do any kind of movements in any order to ensure an individual complete coverage of all parts of both hands [5].

#### Assessment of untreated skin areas

Hands were evaluated under UV light using a Dermalux Box (Bode Chemie GmbH, Hamburg, Germany) before handrub application to make sure that no fluorescent dye was present before the application. Investigators were blinded to treatment type. Evaluation after the application under UV light determined if both were completely covered or if there were any gaps. A subject was categorized as “complete coverage of both hands” if no gaps were found. A subject with an untreated skin area on any hand was defined as a gap in fluorescent dye on the hands, irrespective of location and size. In case of gaps location and size of untreated skin areas were documented with a standard hand drawing. For each application type, the drawings from all 15 subjects were scanned and superimposed for visual assessment of the hand areas with the highest proportion of untreated areas. A darker color indicates a higher frequency of treatment gaps. In addition, a photograph was taken of each hand (palmar and dorsal side) after application for documentation but was not further evaluated.

### Efficacy according to ASTM E 1174-06

Efficacy was determined as described in ASTM E1174-06 with 12 participants per product at Microbiotest, Sterling, USA [12]. Institutional Review Board approval was obtained before enrolling participants (Microbiotest Internal Institutional Review Board, Sterling, VA, USA). All participants’ hands were free from disorders that could have compromised the participant or the study. Participants refrained from using antimicrobials for seven days before the study. A 30-second handwash using nonmedicated soap and a 30-second rinse were performed to remove dirt and oil from hands. The contamination fluid was prepared by inoculating an appropriate volume of tryptic soy broth (TSB) with 0.1 mL of a 24 h culture of *Serratia marcescens* (ATCC 14756) per 100 mL of TSB. This culture was incubated for 24 ± 4 h at 25 ± 2°C. The contamination fluid contained between 5.0 × 10^8^ – 1.0 × 10^9^ colony-forming units (CFU) per mL. Hands were contaminated with 5 mL of the contamination fluid, transferred to hands in three aliquots (1.5, 1.5, and 2 mL) as described in ASTM E 1174-06 [12], and spread over hand surfaces for 45 seconds following each aliquot. After a timed 2-minute air-dry, the glove juice sampling procedure was performed. For each sampling time loose fitting gloves were placed on each hand of the subject within one minute after completing the entire application procedure. A 75 mL aliquot of sampling solution with neutralizers was aseptically added into each glove. The glove of each hand was secured at the wrist and massaged for 1 minute in a uniform manner followed by retrieving a 1 mL aliquot for serial dilution. The first contamination cycle provided baseline population levels. This cycle was followed by a 30-second handwash using nonmedicated soap, a 30-second rinse and a second contamination procedure. Purell Advanced Instant Hand Sanitizer and Purell Advanced Instant Hand Sanitizer Foam were evaluated using an application volume of 1.1 mL, and Sterillium Comfort Gel was evaluated with 2 mL, all as recommended by the manufacturer. Products were rubbed on both hands using the “responsible application” technique until dry. Microbial samples were taken using the glove juice procedure within 1 minute after product application using sampling solution supplemented with valid neutralizing agents (3% polysorbate 80, 0.3% lecithin, 0.1% histidine, 0.1% cysteine). Following the glove juice procedure, an aliquot was removed, diluted in Butterfields Phosphate Buffered Dilution Water containing the same neutralizing agents, and aliquots from the dilution fluid samples were plated onto tryptic soy agar (TSA) containing 0.5% polysorbate 80 and 0.07% lecithin. Plates were incubated at 25°C for approximately 48 hours, red colonies were counted, and log_10_ reductions were calculated. A neutralizer assay was conducted according to ASTM E 1054-08 [13] demonstrating that test products were effectively neutralized (data not shown). It includes determination of the sampling fluid non-toxicity, neutralizer non-toxicity, test material control, and neutralizer effectiveness.

### Efficacy according to ASTM E 2755-10

Efficacy was determined as described in ASTM E 2755-10 with 12 subjects per product at Microbiotest, Sterling, USA [14]. Institutional Review Board approval was obtained before enrolling participants (Microbiotest Internal Institutional Review Board, Sterling, VA, USA). As above, participants’ hands were free from compromising disorders and antimicrobials were not used for seven days before the study. The contamination fluid was prepared by inoculating an appropriate volume of TSB with 1.0 mL a 24 h culture of *Serratia marcescens* (ATCC 14756) per 125 mL of TSB. This culture was incubated for 25 ± 1 h at 35 ± 2°C. The resulting culture contained approximately 1.0 × 10^10^ CFU per mL. It was centrifuged at 7000 G for 10 minutes, the supernatant was decanted, and the pellet re-suspended in TSB to yield a homogenous contamination fluid containing 5.0 × 10^10^ – 1.0 × 10^11^ CFU per mL. The handwash, rinse and *S. marcescens* contamination was as described above, except that 0.2 mL of the contamination fluid was dispensed into the cupped hands, and spread over the hand surfaces for 30 seconds followed by the glove juice sampling procedure to provide the baseline population level. After a second handwash, rinse and second contamination, Purell Advanced Instant Hand Sanitizer, Purell Advanced Instant Hand Sanitizer Foam and Sterillium Comfort Gel were evaluated as described above. Each product was rubbed on both hands until dry using the “responsible application” technique. Microbial samples were taken, reductions were calculated and neutralizer assays were as described above. Test products were effectively neutralized using ASTM E 1054-08 as described above (data not shown).

#### Statistics

Data on hand coverage were analyzed before the applied volume and product identity were unblinded. A chi-square test was used to compare differences in the frequency of incompletely covered hands between volumes for the three products (IBM SPSS Statistics, version 19, Chicago, USA). Two-way ANOVA was applied to determine if the five different volumes or the three different products had a greater impact on the frequency of incompletely covered hands. A p-value < 0.05 was considered to be significant.

## Results

**Figure 1:**
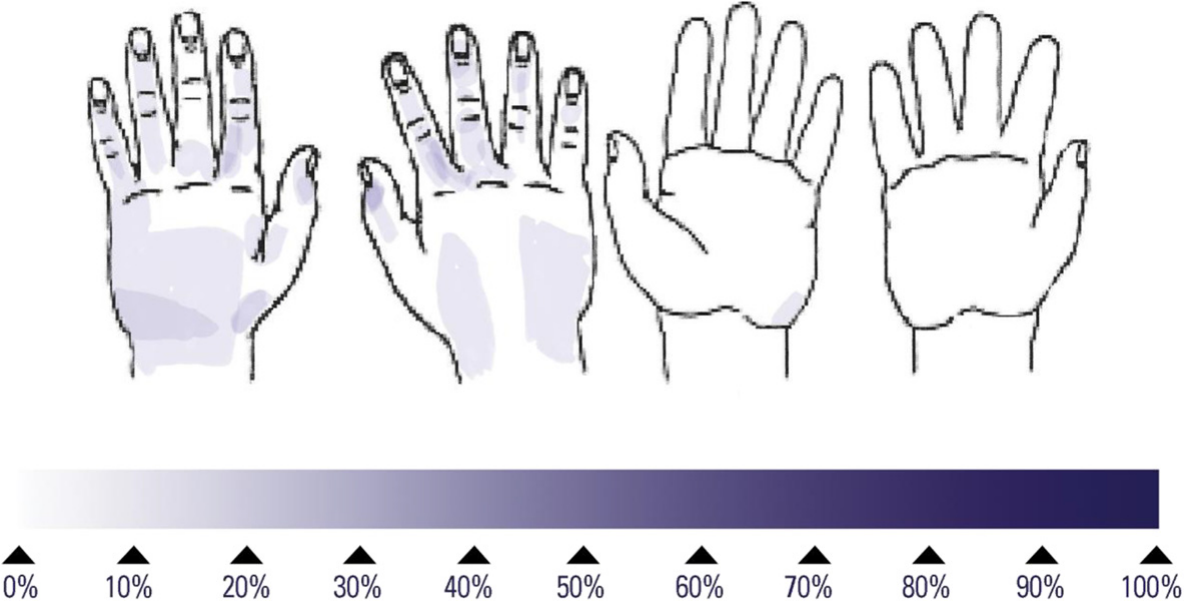
Frequency of untreated skin areas after application of 1.1 mL of Purell Advanced Instant Hand Sanitizer to both hands; darker areas indicate a higher frequency of untreated skin; mean duration obtained with 15 volunteers: 25 seconds.

**Figure 2:**
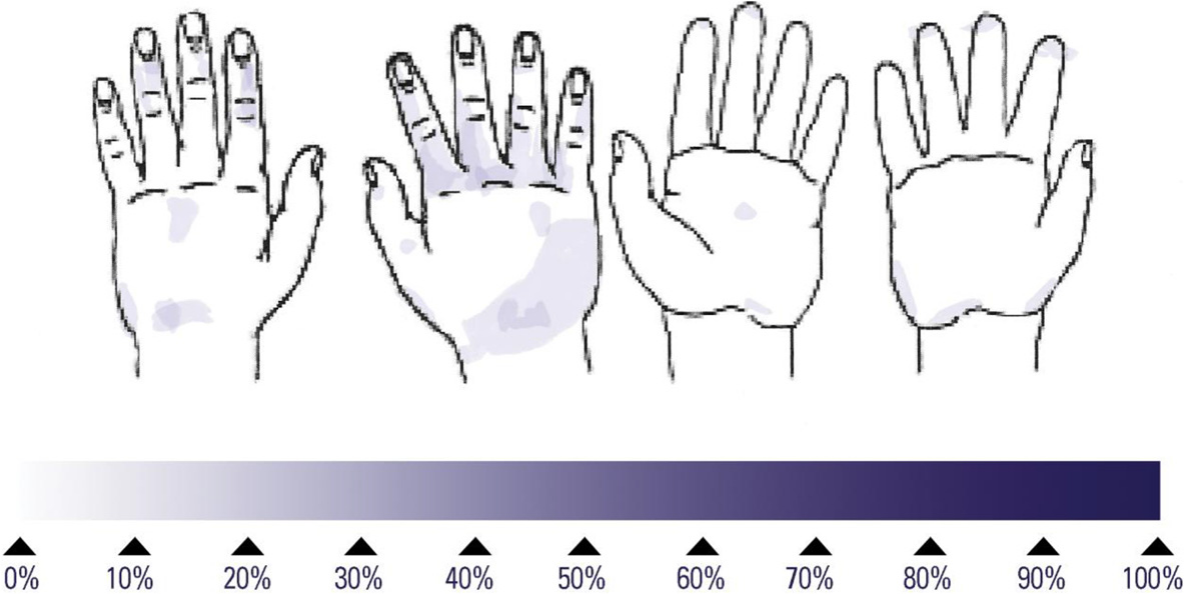
Frequency of untreated skin areas after application of 1.1 mL of Purell Advanced Instant Hand Sanitizer Foam to both hands; darker areas indicate a higher frequency of untreated skin; mean duration obtained with 15 volunteers: 23 seconds.

**Figure 3:**
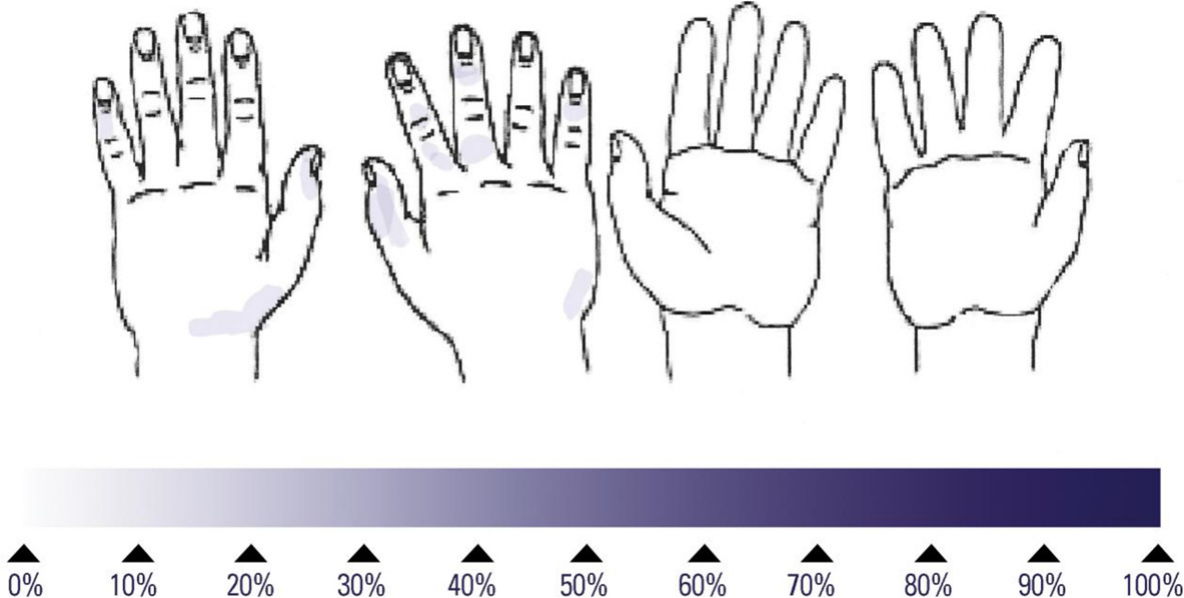
Frequency of untreated skin areas after application of 2 mL of Sterillium Comfort Gel to both hands; darker areas indicate a higher frequency of untreated skin; mean duration obtained with 15 volunteers: 39 seconds.

When small volumes of products were applied (1 pump dispenser push or 1.1 mL), hands took 20 and 29 s to dry (Table 1). Incompletely covered hands were found for 67% of participants who used 1.1 mL and 93% who used single pump dispenser push. Larger volumes (2 mL, 2.4 mL or 2 pump dispenser pushes) required between 34 and 53 s for hands to dry and had better coverage quality, with 0% to 53% incompletely covered hands. Application of small volumes of foam resulted in very poor coverage, with all hands incompletely covered after mean application durations of 6 s (1.1 mL foam), 8 s (2 mL foam) and 11 s (2.4 mL foam). The most common locations of treatment gaps on hands are shown for each hand rub with its recommended volume in Figures 1, 2 and 3. Formulations recommended with 1.1 mL per application show larger untreated areas and more frequent gaps on the back of both hands. No significant differences were seen in the frequency of incompletely covered hands between the three products (p = 0.852; chi-square test); however, differences were significant between the five application volumes used for the three products (p < 0.001; chi-square test). Two-way ANOVA showed that product type had no significant influence on the frequency of incompletely covered hands (p = 0.796) but the applied volume did (p < 0.001).

**Table 1:** Mean duration of hand rub procedure until hands feel completely dry and frequency of incompletely covered hands

	**1.1 ml product**		**1 pump dispenser push of product**			**2 ml product**		**2.4 ml product**		**2 pump dispenser pushes of product**		
**Product**	**Time**	**Leaks**	**Time**	**Leaks**	**Applied volume (mean)**	**Time**	**Leaks**	**Time**	**Leaks**	**Time**	**Leaks**	**Applied volume (mean)**
Purell Advanced Instant Hand Sanitizer	25 s	73%	28 s	80%	1.3 mL	37 s	27%	46 s	13%	53 s	47%	2.7 mL
Purell Advanced Instant Hand Sanitizer Foam	23 s	67%	20 s	87%	0.7 mL	41 s	40%	49 s	13%	34 s	33%	1.5 mL
Sterillium Comfort Gel	20 s	87%	29 s	93%	1.6 mL	39 s	53%	41 s	27%	51 s	0%	3.1 mL

Data from 15 subjects per procedure were obtained with 3 alcohol-based hand rubs applied in 5 different amounts.

Application of 1.1 mL of 70% (v/v) ethanol-based products did not meet the minimum efficacy requirement of a 2 log_10_-reduction as outlined by the FDA [9] in both ASTM E 1174-06 and ASTM E 2755-10 (Table 2). Application of 2 mL of the 85% (w/w) ethanol-based product, however, showed a 2.90 log_10_-reduction according to ASTM E 2755 and a 2.06 log_10_-reduction according to ASTM E 1174-06.

**Table 2:** **Mean log**_**10**_**-reduction of *Serratia marcescens *with three commercially available hand rubs applied as recommended by the manufacturer**

**Type of hand rub**	**Applied volume as recommended by manufacturer**	**Mean log**_**10**_**-reduction (ASTM E 2755-10)**	**Mean log**_**10**_**-reduction (ASTM E 1174-06)**
Sterillium Comfort Gel	2 ml	2.90 ± 0.33	2.06 ± 0.33
Purell Advanced Instant Hand Sanitizer	1.1 ml	1.97 ± 0.45	1.85 ± 0.60
Purell Advanced Instant Hand Sanitizer Foam	1.1 ml	1.96 ± 0.31	1.60 ± 0.55

Data were obtained according to ASTM E 2755-10 and ASTM E 1174-06 with 12 subjects.

## Discussion

Our data indicated that the trend towards using smaller volumes such as 1.1 mL for hand disinfection could lead to substantial risks such as incompletely covered hands and noticeably low efficacy. WHO does not provide efficacy criteria to be fulfilled (e.g., EN 1500), or a recommended minimum volume per application. The WHO recommendation is to “cover all surfaces of the hands” but a single pump dispenser push or 1.1 mL does not achieve this goal.

Of note, the two 70% (v/v) ethanol-based rubs applied with 1.1 mL did not meet the US FDA efficacy requirement of a 2 log_10_-reduction after the first application. Application of 2 mL of 85% (w/w) ethanol hand gel did. This finding adds relevant information to previously published data on the same disinfectant formulations. Edmonds et al. [8] described two formulations based on 70% (v/v) alcohol that meet the 2 log_10_-efficacy requirement according to ASTM E 1174 but with volumes of 5 and 2 mL. Our data raise doubts that a volume of 1.1 mL of the same formulations has sufficient antimicrobial activity. In addition, data in Edmonds et al. [8] were collected with an outdated 1994 version of ASTM E 1174 instead of the updated 2006 version (Table 3). Both the lack of neutralizing agents in the sampling fluid [15] and the lack of a maximum time between product application and sampling make it likely that the measured log_10_-reduction was an overestimation of efficacy on hands [16]. According to EN 1500, two handrubs based on 73%–78% (w/w) alcohol failed the efficacy requirement when applied with 2 mL for 30 s [17]. Based on our data, 1.1 mL, in addition to poor quality of hand coverage, also provides insufficient antimicrobial activity.

**Table 3:** Evaluation of efficacy data obtained with three hand rubs according to different versions of ASTM E 1174

**Product**	**Volume**	**Version of ASTM E 1174**	**Mean log**_**10**_**-reduction**	**Neutralization in sampling fluid including validation**	**Time between end of application and sampling**	**Likelihood of false positive data**	**Meets US FDA requirements**	**Complete hand coverage**
Purell Advanced Instant Hand Sanitizer	5 ml	1994	3.58*	No	No maximum	High	Yes	Likely
	2 ml	1994	3.35*	No	No maximum	High	Yes	Likely
	1.1 ml	2006	1.85**	Yes	Maximum: 60 s	Very low	No	Unlikely
Purell Advanced Instant Hand Sanitizer Foam	5 ml	1994	3.55*	No	No maximum	High	Yes	Likely
	2 ml	1994	3.48*	No	No maximum	High	Yes	Likely
	1.1 ml	2006	1.60**	Yes	Maximum: 60 s	Very low	No	Unlikely
Sterillium Comfort Gel	5 ml	1994	3.12*	No	No maximum	High	Yes	Likely
	2 ml	2006	2.06**	Yes	Maximum: 60 s	Very low	Yes	Likely

Methodological differences may explain conflicting results. *Data described by Edmonds et al. 2012 [8]; **data coming from this study.

It is also interesting to notice that the mean log_10_ reduction obtained with three different ethanol-based hand rubs was slightly higher when determined according to ASTM E 2755 compared to ASTM E 1174. A possible explanation is the volume of contamination fluid which is 0.2 mL according to ASTM E 2755 and 5 mL according to ASTM E 1174. The smaller amount of broth on both hands may well explain the slightly higher efficacy because ethanol is known to exhibit a non-specific mode of antimicrobial activity by protein denaturation [18].

We also observed an obvious correlation between applied volumes and the corresponding contact time on hands. This finding confirms previously published data by Cheeseman et al. [19] who reported that with a specific gel one pump from a dispenser may require only 9 s to rub until dry whereas two pumps require 27 s. A shorter application time is also associated with a lower antimicrobial activity [3] so that a sufficient amount of hand rub seems mandatory to ensure complete hand coverage for the recommended application time.

Our participants used a “responsible application” technique to ensure complete coverage of both hands, as described in [5]. In a previous study, application of 3 mL of handrub with the same technique showed 53% to 55% incomplete coverage of hands whereas application by the six steps of EN 1500 yielded worse results (67%–100%) [5]. In our study, incomplete coverage rates were lower, but only for volumes of 2 mL or more; specifically, rates were 40% with 2 mL, 18% with 2.4 mL, and 27% with 2 dispenser pushes. Our data indicated that the responsible application technique has a better potential to ensure optimum hand coverage than the six steps of EN 1500. In Germany, “responsible application” has been recommended since 2011 by the national WHO campaign (“Aktion Saubere Hände”) [20]. The observed coverage rates indicate that the WHO recommendation “cover all surfaces of the hands” is not easily fulfilled, especially with small volumes such as 1.1 mL. The “responsible application technique” we used in our study seemed to be the best possible technique to ensure optimum coverage. Using other techniques such as the six steps of EN 1500 or a volume < 2 mL are likely to jeopardize effectiveness goals.

## Conclusions

Our data indicated that handrubs based on 70% ethanol (v/v) used as recommended with 1.1 mL per application are not suitable to ensure complete coverage of both hands and do not fulfill the current ASTM E 1174 and ASTM E 2755 efficacy standards.

## Competing interests

The first four authors are paid employees of Bode Chemie GmbH, Hamburg, Germany.

## Authors’ contributions

GK, SE and NN designed the study, SR organized and supervised all experiments on hand coverage, MB organized and supervised all experiments on efficacy, GK analyzed the data and wrote the manuscript, all authors read and approved the final manuscript.

## Pre-publication history

The pre-publication history for this paper can be accessed here:

http://www.biomedcentral.com/1471-2334/13/472/prepub

## References

[B1] Anonym WHO guidelines on hand hygiene in health care. First global patient safety challenge clean care is safer care 2009 Geneva: WHO 23805438

[B2] Rotter M Skopec M Kampf G Entwicklung der Händehygiene und die Bedeutung der Erkenntnisse von Ignaz Ph. Semmelweis Hände-hygiene im gesundheitswesen 2003 Berlin: Springer 1 27

[B3] Dharan S Hugonnet S Sax H Pittet D Comparison of waterless hand antisepsis agents at short application times: raising the flag of concern Infect Contr Hosp Epidemiol 2003 24 3 160 164 10.1086/502182 12683505

[B4] EN 1500:1997 Chemical disinfectants and antiseptics. Hygienic hand disinfection. Test method and requirement (phase 2, step 2) 1997 Brussels: CEN–Comité Européen de Normalisation

[B5] Kampf G Reichel M Feil Y Eggerstedt S Kaulfers P-M Influence of rub-in technique on required application time and hand coverage in hygienic hand disinfection BMC Infect Dis 2008 8 149 10.1186/1471-2334-8-149 18959788 PMC2600642

[B6] Kramer A Rudolph P Kampf G Pittet D Limited efficacy of alcohol-based hand gels Lancet 2002 359 1489 1490 10.1016/S0140-6736(02)08426-X 11988252

[B7] Kampf G Marschall S Eggerstedt S Ostermeyer C Efficacy of ethanol-based hand foams using clinically relevant amounts: a cross-over controlled study among healthy volunteers BMC Infect Dis 2010 10 78 10.1186/1471-2334-10-78 20338067 PMC2864273

[B8] Edmonds SL Macinga DR Mays-Suko P Duley C Rutter J Jarvis WR Arbogast JW Comparative efficacy of commercially available alcohol-based hand rubs and world health organization-recommended hand rubs: formulation matters Am J Infect Contr 2012 40 6 521 525 10.1016/j.ajic.2011.08.016 22264743

[B9] Anonymous Tentative final monograph for health care antiseptic products; proposed rule Federal Reg 1994 59 116 31401 31452

[B10] Kampf G How effective are hand antiseptics for the post-contamination treatment of hands when used as recommended? Am J Infect Contr 2008 36 5 356 360 10.1016/j.ajic.2007.07.017 18538702

[B11] Edmonds S Macinga DR Paulson D The influence of ABHR product format on *in vivo* efficacy: a meta-analysis Am J Infect Contr 2012 40 5 e43

[B12] American Society for Testing and Materials International ASTM E 1174-06: Standard test method for evaluation of the effectiveness of healthcare personnel handwash formulations 2006 West Conshohocken, PA: American Society for Testing and Materials

[B13] American Society for Testing and Materials International ASTM E 1054-08: standard test methods for evaluation of inactivators of antimicrobial agents 2008 West Conshohocken, PA: American Society for Testing and Materials

[B14] American Society for Testing and Materials International ASTM E 2755-10: standard test method for determining the bacteria-eliminating effectiveness of hand sanitizer formulations using hands of adults 2010 West Conshohocken, PA: American Society for Testing and Materials

[B15] Kampf G Shaffer M Hunte C Insufficient neutralization in testing a chlorhexidin-containing ethanol-based hand rub can result in a false positive efficacy assessment BMC Infect Dis 2005 5 48 10.1186/1471-2334-5-48 15963239 PMC1181814

[B16] Eggerstedt S Comparative efficacy of commercially available alcohol-based hand rubs and world health organization-recommended hand rubs Am J Infect Contr 2013 41 5 472 474 10.1016/j.ajic.2013.01.021 23622705

[B17] Goroncy-Bermes P Koburger T Meyer B Impact of the amount of hand rub applied in hygienic hand disinfection on the reduction of microbial counts on hands J Hosp Infect 2010 74 3 212 218 10.1016/j.jhin.2009.09.018 20061058

[B18] Kampf G Kramer A Epidemiologic background of hand hygiene and evaluation of the most important agents for scrubs and rubs Clin Microbiol Rev 2004 17 4 863 893 10.1128/CMR.17.4.863-893.2004 15489352 PMC523567

[B19] Cheeseman KE Denyer SP Hosein IK Williams GJ Maillard JY Evaluation of the bactericidal efficacy of three different alcohol hand rubs against 57 clinical isolates of S. aureus J Hosp Infect 2009 72 4 319 325 10.1016/j.jhin.2009.04.018 19596492

[B20] Wissenschaftlicher Beirat der Aktion Saubere Hände Positionspapier “Einreibemethode” 2011 http://www.aktion-sauberehaende.de/downloads/pdf/ASH_Positionspapier_Einreibemethode_30092011.pdf

